# Supraclavicular lymph node metastasis in esophageal carcinoma: a topic of ongoing controversy

**DOI:** 10.3389/fonc.2025.1527625

**Published:** 2025-01-27

**Authors:** Bowen Zhang, Huan Zhang, Yu Chen, Wanli Xia, Yichun Wang

**Affiliations:** ^1^ Department of Radiation Oncology, The First Affiliated Hospital of Anhui Medical University, Hefei, Anhui, China; ^2^ Department of Thoracic Surgery, The First Affiliated Hospital of Anhui Medical University, Hefei, Anhui, China

**Keywords:** esophageal cancer, lymphatic metastasis, cancer staging, prognosis, supraclavicular lymph node

## Abstract

Lymph node metastasis is an important prognostic factor in esophageal carcinoma (EC). Currently, there are no consensus-based staging methods for EC with supraclavicular lymph node (SCLN) metastasis. In this review, we present a summary of several classification methods of the SCLNs and highlight their differences in anatomic definitions. Then, we analyze the lymphatic drainage of the SCLNs from esophagus and the distribution patterns of the SCLNs metastasis from EC. Moreover, we discuss the prognostic influence and different staging methods of the SCLN metastasis. In summary, the variations in different classification methods make the SCLNs confusing for clinical application. A standardized and precise definition of the SCLNs should be established urgently for EC. SCLNs can drain lymphatics at various levels of the esophagus, even from the intramural esophagus directly. Therefore, the SCLNs can be metastatic in superficial EC and even become sentinel nodes. Metastatic SCLNs are usually located on the surface of the scalenus anterior muscle and near the venous angle. Increasing pieces of evidence have shown that patients with SCLN metastasis have similar survival than those with regional lymph node metastasis and better survival than those with organ metastasis, which bring challenges to current staging methods.

## 1 Introduction

Esophageal carcinoma (EC) is the seventh leading cause of cancer death worldwide in 2022 (1). Lymphatic spread of cancer cells is common, and lymph node metastasis can be present widely from neck to abdomen in EC, even in patients with early stages (2). The regional lymph node status is considered as a reliable predictor of survival in EC. The American Joint Committee on Cancer (AJCC) staging manual and the Japanese Classification of Esophageal Cancer published by Japan Esophageal Society (JES) are two widely used staging systems in EC. However, the two have great differences in the nodal staging, especially for patients with supraclavicular lymph node (SCLN) metastasis (3–8). One controversial topic is whether the SCLN metastasis belongs to distant metastasis or regional metastasis.

The accurate prognostic assessment of SCLN metastasis in EC is particularly important. A number of studies have shown that the presence of SCLN metastasis did not have a significant impact on overall survival (OS), and it should be classified as regional lymph node (9, 10). However, other studies have shown different results and suggested that SCLN metastasis should be classified as distant metastasis in EC (11, 12). The lack of consensus-based results leads to great discrepancies in staging and treatment strategies for EC with SCLN metastasis. In the present review, we will discuss the classification methods, lymphatic drainage of esophageal layers, and metastatic patterns of SCLN, then retrieve relevant literature to discuss the disputes on the prognosis of SCLN metastasis in EC. This will help us better understand the SCLN metastasis and resolve the discrepancies of EC in future studies.

## 2 Classification of SCLNs

**Figure 1:**
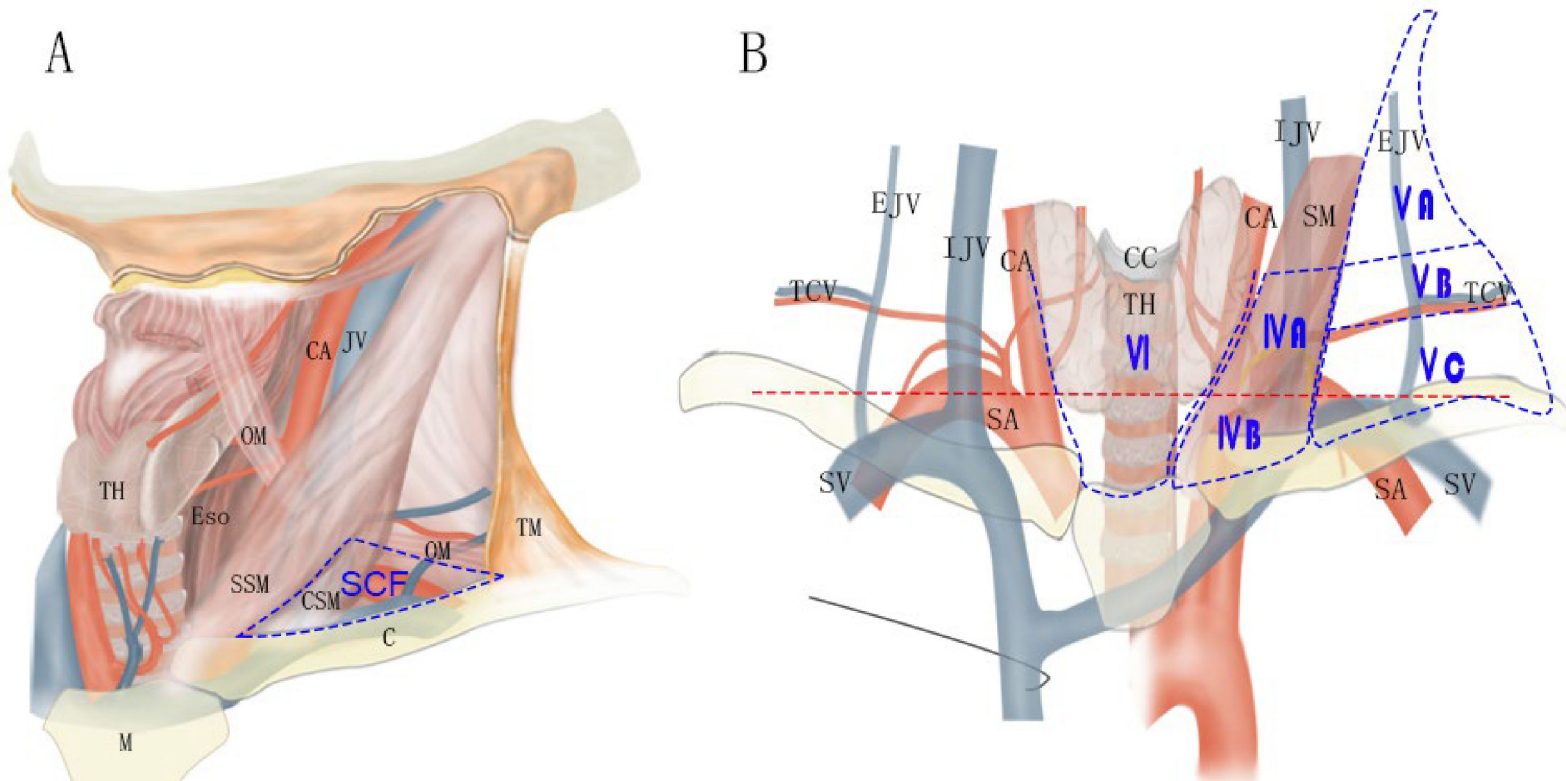
Schematic indicating the location of SCF **(A)** and the lymph node levels in lower neck according to the 2013 International Cervical Node Level Consensus **(B)**. C, clavicle; CA, common carotid artery; CC, cricoid cartilage; CSM, clavicular head of sternocleidomastoid muscle; EJV, external jugular vein; Eso, esophagus; IJV, internal jugular vein; M, manubrium; OM, omohyoid muscle; SM, sternocleidomastoid muscle; SSM, sternal head of sternocleidomastoid muscle; TCV, transversal cervical vessels; TH, thyroid gland; TM, trapezius muscle. Red line: the level of 2 cm above the sternal notch.

Anatomically, SCLNs are usually lymph nodes embedded in the supraclavicular fossa (SCF) and part of the cervical lymph nodes. Broadly speaking, the SCF consists of a lesser fossa and a greater fossa in the lower neck (**Figure 1A**). The lesser SCF is a depression between the sternal and clavicular heads of the sternocleidomastoid muscle. The greater SCF refers to the shallow depression that overlies the supraclavicular triangle, which is formed by the sternocleidomastoid muscle, inferior belly of the omohyoid muscle, and clavicle (13).

In clinical practice, there are several methods to classify the SCLNs, which make them confusing for application. The 2013 International Cervical Node Level Consensus (ICNLC) has defined node levels of the SCLNs from the perspective of head and neck cancers (14). In this consensus, the SCLNs are divided into two groups: sublevel IVb (medial supraclavicular group) and sublevel Vc (lateral supraclavicular group) (**Figure 1B**). The International Association for the Study of Lung Cancer (IASLC) also established a new lymph node map in 2009 (15). In this map, the SCLNs belong to lymph node station 1 (level 1), which also includes the lower cervical and sternal nodes. In the sixth and seventh editions of the AJCC cancer staging manual, the SCLNs adapted the same definition of the 2009 IASLC lymph node map (6, 7). However, in the latest eighth edition of the AJCC staging manual (8), the nomenclature of cervical regional lymph nodes follows that of head and neck in this manual and the sublevel Vb includes lymph nodes following the transverse cervical vessels and the SCLNs. In the JES staging system (3–5), the SCLNs belong to station 104, which are located in the supraclavicular fossa, extending from the lower border of the cricoid cartilage superiorly, to the clavicle inferiorly, including the lower internal deep cervical lymph nodes. The medial boundary is the medial border of the carotid sheath. As shown in **Table 1**, these classification methods have many variations with regard to levels, locations, and anatomical boundaries of the SCLNs. Moreover, the anatomical boundaries of the SCLNs are not clearly defined in many classification methods. For example, the SCLNs are not distinguished as a separate group in the 2009 IASLC map and the eighth edition of the AJCC staging manual, and the external boundary of station 104 is not explained in the JES staging system. Therefore, a unified and precise classification method of the cervical lymph nodes for EC should be established in the future.

**Table 1:** Different classifications of the SLNs.

Methods	Level/station	Corresponding level (2013 ICNLC)	Name of lymph nodes
2013 ICNLC	IVb Vc	IVb Vc	Medial SCLNs Lateral SCLNs
2009 IASLC, 6th and 7th AJCC	1	IVa, IVb, Vb, Vc, lower part of VIa and VIb	SCLNs, lower cervical and sternal notch nodes
8th AJCC	Vb	Vb, and Vc	SCLNs and transverse cervical vessel nodes
JES	104	IVa, IVb, Vb, and Vc	SCLNs

AJCC, American Joint Committee on Cancer; IASLC, International Association for the Study of Lung Cancer; ICNLC, International Cervical Node Level Consensus; JES, Japan Esophageal Society; SCLN, supraclavicular lymph node.

## 3 Lymphatic drainage of esophagus in the supraclavicular region

Before emptying to the venous circulation, the lymphatics are usually collected by the right lymphatic duct and the thoracic duct on the left (16). In the supraclavicular region, the deep cervical lymph nodes situated on the scalenus anterior usually have direct connections with the terminal tributaries of the thoracic duct or the right lymphatic duct (17). Moreover, these nodes can collect the lymphatics from the internal jugular trunk, the subclavian trunk, and the bronchomediastinal trunk. Therefore, these deep cervical lymph nodes may become a major metastatic target of tumor cells spread in many cancers.

**Figure 2:**
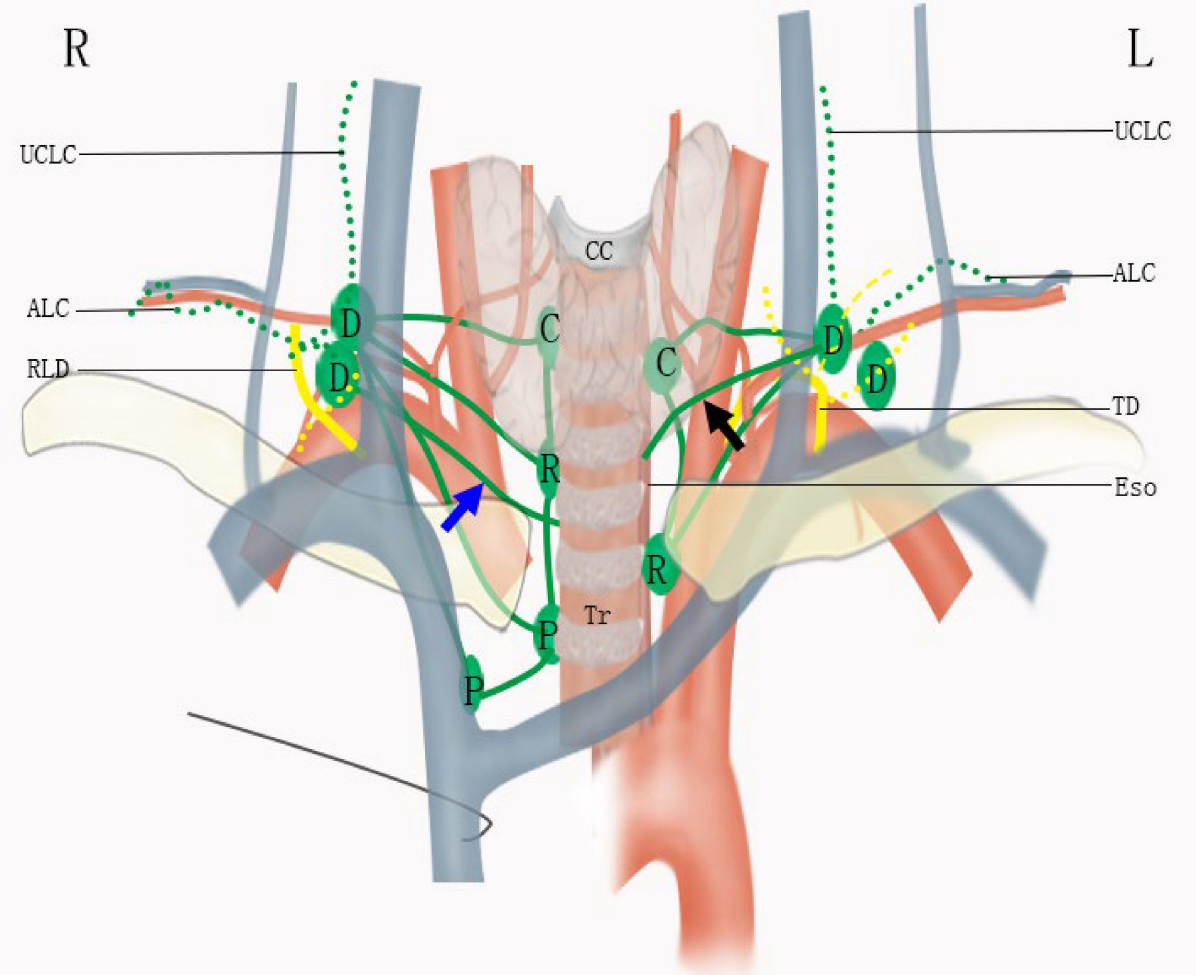
Lymphatic drainage of the esophagus in the lower neck. ALC, accessory lymphatic chain; C, cervical paraesophageal lymph node; CC, cricoid cartilage; D, deep cervical lymph node; Eso, esophagus; P, paratracheal lymph node; R, recurrent nervous lymph node; RLD, right lymphatic duct; TD, thoracic duct; Tr, trachea; UCLC, upper cervical lymphatic chain. The blue and black arrows indicate direct drainage from esophagus to deep cervical lymph nodes.

The esophagus has a complex lymphatic drainage system, characterized by longitudinal lymphatic vessels in the submucosa and their direct drainage to extramural lymph nodes (2, 18). As a result, extensive lymph node metastasis from the cervical to the abdominal region is common in EC, even in early-stage diseases. In the lower neck, the deep cervical lymph nodes are usually the upmost nodes for thoracic esophagus and often involved by metastasis in EC. On the contrary, the upper cervical lymphatic chain and the accessory lymphatic chain does not receive lymphatic from the esophagus except for lymph reflux in special circumstances. Therefore, lymph nodes in these regions are rarely involved, except in very advanced diseases. The lymphatic relay of the deep cervical lymph nodes in the lower neck for esophagus is shown in **Figure 2**.

On the right, the ascending paratracheal lymphatic chain is well-developed. Lymph vessels can ascend to reach the recurrent laryngeal lymph nodes and cervical paraesophageal lymph nodes or run on the subserous surface of the mediastinal pleura under the subclavian artery, then run over the artery and the scalenus anterior muscle to reach the deep cervical nodes. The recurrent laryngeal lymph nodes and cervical paraesophageal lymph nodes drain into the venous angle with or without the joint of the deep cervical nodes (2, 18). Moreover, the lymphatics from the intramural esophagus can drain directly to the right deep cervical nodes (2, 18), as shown in **Figure 2** with a blue arrow.

Due to the presence of the thoracic duct, the left ascending paratracheal lymphatic chain is poorly developed. Lymphatics from mediastinum can empty into the thoracic duct directly, and their joints to the deep cervical nodes are fewer on the left (2). However, the left deep cervical nodes have consistent communications with the terminal tributaries of the thoracic duct. Same as the Virchow’s metastasis in gastric cancer (19), this lymph reflux from the terminal parts of the thoracic duct can also result in left deep cervical lymph nodes metastasis in EC. Additionally, the intramural lymphatic from the esophagus can also be drained directly by the left deep cervical nodes, as shown in **Figure 2** with a black arrow (18).

In summary, although the lower deep cervical lymph nodes are dispensable relay routes, they can be the last stations on the way of esophageal lymphatic drainage before the venous circulation. They not only drain lymphatics from lymph nodes in the lower neck and upper mediastinum but also can connect directly the intramural lymphatics of the esophagus and the terminal parts of the thoracic duct or the right lymphatic duct. Because of this complex lymphatic drainage, it is difficult to determine the appropriate nodal staging for EC patients with metastasis in these nodes. They can be regional lymph nodes for the esophagus, even the first station sometimes. However, lymph nodes near the accessory lymphatic chain are out of the regular route and should be distinguished from these deep cervical lymph nodes.

## 4 Patterns of SCLN metastasis in EC

The unilateral SCLN metastasis rate is 3%–10% for EC that underwent upfront surgery, and the right SCLNs usually have higher metastasis rates than the left SCLNs except for lower thoracic EC (2, 20–22). A possible explanation is the well-developed right paratracheal lymphatic chain, which results in more joints with the right SCLNs. In our previous study (2), we found that the metastasis rate of the left SCLNs decreased insignificantly when the primary tumor site descended from the upper esophagus to the lower esophagus compared to that of the right SCLNs. Except the route of the ascending mediastinal lymphatic chain without the thoracic duct, the thoracic duct is another important route for the metastatic involvement of left SCLNs in EC. As the primary site of the tumor descends, the SCLN metastasis through the ascending mediastinal lymphatic chain will decrease while the left SCLN metastasis may increase through the route of thoracic duct caused by lymph reflux, using the same way of Virchow’s metastasis in gastric cancer.

In superficial EC invading muscularis mucosa and submucosa, the SCLNs with or without other regional lymph nodes can be involved (23, 24). Sentinel node mapping has indicated that the SCLNs were also frequently identified in the cervical area for the upper and middle thoracic EC (25, 26). These results verify the above-mentioned anatomical characteristics that the SCLNs may become the early metastatic stations. Therefore, the SCLN metastasis may not be an indicator of advanced disease in many patients.

Recently, several studies have analyzed the anatomic distribution patterns of the lower cervical lymph node metastasis in EC using imaging techniques, such as computed tomography (CT). It can be seen that these metastatic lymph nodes affect mainly the cervical tracheoesophageal nodes and medial SCLNs located on the surface of the scalenus anterior muscle, mediastinal pleura, and subclavian artery (27–31). This metastatic pattern is also present in small cell lung cancer (32). However, it is different from cancers of the head and neck, in which lymphatic metastasis tends to spread mainly to the region lateral to the carotid sheath and rarely to the tracheoesophageal nodes (29). Additionally, the metastatic SCLNs in EC are usually in the lower part of the supraclavicular region, particularly close to the venous angle. It was found that the distance between these SCLNs and the venous angle was usually <2.0 cm (29), which is very close to the distance between the apex of the thoracic duct and its end point and the length of the right lymphatic duct (16, 33).

## 5 Prognosis and staging of SCLN metastasis in EC

### 5.1 Literature search

A systematic search was conducted in PubMed, Scopus, and Web of Science to identify articles on prognosis of the SCLNs for esophageal cancer reported before July 21, 2024. Keywords included “esophageal cancer” and “supraclavicular lymph node.” The complete search strategy is available in the **Supplementary Material**.

### 5.2 With or without SCLN metastasis

As shown in **Table 2**, several retrospective studies have evaluated the association of the SCLN metastasis status and the survival in EC that underwent definitive chemoradiotherapy or esophagectomy with lymphadenectomy. Most studies have shown that clinical SCLN metastasis was not a poor prognosis factor for patients who received definitive chemoradiotherapy (34–37). In another study, although the SCLN metastasis had a much poorer survival in all population, it was suggested that clinical SCLN metastasis should be considered to be regional lymph nodes for cervical or upper thoracic EC and a higher N stage or M1 stage for the middle or lower thoracic EC (38). For patients who underwent esophagectomy, two studies showed that there was no significant difference in survival between patients with SCLN metastasis and those without (9, 39). Many studies showed that patients with SCLN metastasis had poor survival (10–12, 40, 41). However, the SCLN metastasis was not always an independent prognostic factor (10). Furthermore, one study showed that there was no statistically significant difference in survival between patients with SCLN metastasis before chemotherapy but not after therapy and patients free of SCLN metastasis before and after chemotherapy (40).

**Table 2:** Studies evaluating the prognostic value of SCLN metastasis in EC.

First Author (year)	Time period	Stage no.	SCLN definition	Treatments	Groups	Number	SCC/others	U/M/L	OS	p-value
JH Yen 2020 (34)	2006–2017	cT1-4N1-3	Not reported	dCRT	SCLN (+)	71	71/0	24/33/14	5-year: 11.3%	0.88
					SCLN (−)	72	72/0	23/22/27	5-year: 15.2%	
X Li 2017 (35)	2008–2013	cT1-4N1-3	IASLC Level 1	dCRT/dRT	SCLN (+)	174	174/0	70/57/47	mOS: 19m	0.785
					SCLN (−)	119	119/0	30/51/38	mOS: 17m	
PM Jeene 2016 (36)	2003–2013	cT1-4N0-3	LNs in SCF	dCRT	SCLN (+)	37	–	–	mOS: 23.6 m	0.51
					SCLN (−)	160	–	–	mOS: 17.1 m	
YH Chen 2018 (37)	2000–2015	cT1-4N0-3	LNs in SCF	dCRT	SCLN (+)	70	70/0	–	–	0.28
					SCLN (−)	299	299/0	–	–	
HY Xu 2018 (38)	2009–2015	cT1-4N0-3	IASLC Level 1	dCRT	SCLN (+)	155	143/12	45/88/22	5-year: 18.5%	<0.001
					SCLN (−)	596	567/29	158/332/106	5-year: 25.1%	
S Kosugi 2013 (39)	2002–2011	pT1-2N0-3	JES station 104	S with 3FL	SCLN (+)	6	–	2/4/0	5-year:46.2%	0.06
					SCLN (−)	80	–	15/55/10	5-year:77.8%	
SY Park 2023 (9)	1994–2018	pT1-4N0-3	LNs in SCF	S with 3FL	SCLN (−) ※	287	287/0	–	5-year:41.5%	0.054
					SCLN (+) ※	75	75/0	–	5-year:25.6%	
Y Tachimori 2014 (10)	2001–2003	pT0-4a N0-3	JES station 104	S with 3FL	SCLN (−) ※	560	–	–	5-year:40.4%	<0.001
					SCLN (+) ※	190	–	–	5-year:24.1%	
W. Hu 2014 (11)	2000–2008	pTis-4aNx	IASLC Level 1	S with 3FL	SCLN (+)	72	72/0	53/16/3	5-year:24.0%	<0.001
					SCLN (−)	204	204/0	115/60/29	5-year:59.2%	
Y Numata 2021 (12)	2008–2018	pT1-4N0-3	JES station 104	S with LD	SCLN (+) ※	17	16/1	17/0/0	5-year:7.2%	<0.001
					SCLN (−) ※	27	25/2	27/0/0	5-year:46.2%	
H Miyata 2015 (40)	2000–2011	pT0-4 N0-3	Not reported	NCT + S with 2FL/3FL	SCLN (−) ※	169	–	–	3-year:47.5%	0.003
					SCLN (+) ※	47	–	–	3-year:20.1%	
FD Wang 2019 (41)	2014–2017	pT1-4 N0-3	Not reported	S with 3FL	SCLN (+)	35	35/0	–	mOS: 21.0 m	<0.001
					SCLN (−)	128	128/0	–	mOS: 39.0m	

dCRT, definitive chemoradiotherapy; dRT, definitive radiotherapy; IASLC, International Association for the Study of Lung Cancer; JES, Japan Esophageal Society; L, lower; LD, lymph node dissection; M, middle; mOS, median overall survival; NCT+S, neoadjuvant chemotherapy followed by surgery; OS, overall survival; S, surgery; SCC, squamous cell carcinoma; SCF, supraclavicular fossa; SCLN, supraclavicular lymph node; U, upper; 2FL, 2-fields lymphadenectomy; 3FL, 3-fields lymphadenectomy**;** ※patients with positive regional lymph node. # the American Joint Committee on Cancer Staging Manual (seventh or eighth edition) was used for the staging classification.

### 5.3 Metastasis in the SCLNs versus others

Retrospective studies have shown that patients with SCLN metastasis had a similar survival compared to patients with metastasis in other regional lymph nodes, such as cervical paraesophageal lymph nodes (42, 43). The same results were also found in studies comparing metastasis in cervical lymph nodes or SCLNs with metastasis in mediastinal or abdominal lymph nodes (44), or stage III (45), or M0 stage (46). In another study, patients with SCLN metastasis had even better 5-year OS than those with cervical paraesophageal lymph node metastasis (34% vs. 21%, p=0.0416) (47). However, numerous studies have shown that patients with distant metastasis had a significantly poor survival than patients with SCLN metastasis in EC (34, 44, 48–50). It was recommended that the SCLNs should be reclassified as regional lymph nodes, and the SCLN metastasis can be considered as N2 stage for thoracic EC (51).

### 5.4 Influence of the primary sites

In the JES staging system, the nodal stage is classified by the locations of regional lymph nodes and the primary sites of EC. A large retrospective Japanese study indicated that the survival difference was not significant in the upper EC but significant in the middle or lower EC between node-positive patients without SCLN metastasis and node-positive patients with SCLN metastasis (10). Another study showed that there was no survival difference between patients with SCLN metastasis and patients without SCLN metastasis in all EC (35). Multivariate analyses based on patients from the Surveillance Epidemiology and End Results (SEER) database indicated that the SCLN metastasis was an independent prognostic factor in the lower thoracic EC but not in the upper thoracic EC (52), which was similar to Wang’s results (41). Therefore, it seems that SCLNs should be considered as regional lymph nodes for proximal EC and higher N stage or M1 stage for distal EC. Additionally, SCLNs dissection are usually recommended for surgical treatment of proximal EC and selected middle and distal EC (53).

### 5.5 Staging of EC with SCLN

In the AJCC staging manual and the JES staging system, there are great differences for EC with SCLN metastasis, even in different editions of the same staging system, as shown in **Table 3**. Prior to the 12th edition of the JES staging system, SCLNs were considered as regional lymph nodes. However, they were classified as distant lymph node metastasis (M1a) in the latest 12th edition. It is well-known that SCLNs were considered as M1a for upper thoracic EC and M1b (non-regional lymph node metastasis and/or other distant metastasis) for middle or lower EC in the sixth edition of the AJCC staging manual. However, SCLNs were classified as regional lymph nodes in the seventh AJCC staging manual but M1 (distant metastasis) in the eighth edition for EC.

**Table 3:** Different staging methods for EC with SCLN metastasis.

	10th JES (3)	11th JES (4)	12th JES (5)	6th AJCC (6)	7th AJCC (7)	8th AJCC (8)
Ce	N2	N2	Regional	Regional	Regional	M1
Ut	N2	N2	M1a	M1a	Regional	M1
Mt	N3	N2	M1a	M1b	Regional	M1
Lt	N4/M1a	N3	M1a	M1b	Regional	M1

AJCC, American Joint Committee on Cancer; Ce, cervical esophageal cancer; JES, Japan Esophageal Society; Lt, lower thoracic esophageal cancer; Mt, middle thoracic esophageal cancer; Ut, upper thoracic esophageal cancer.

In summary, the prognostic impact of the SCLN metastasis in EC has been questioned for many years. Most of the previous studies have shown that SCLN metastasis was not an independent prognostic factor. Patients with SCLN metastasis seem to have similar survival to those with regional lymph node metastasis and better survival than those with organ metastasis. Furthermore, the influence of SCLN metastasis may change in EC with different primary sites. We should also note that although SCLN metastasis may be a poor prognostic factor in many studies, it cannot be simply defined as an indicator of advanced diseases in EC. These studies bring challenges to current staging methods of SCLN metastasis in EC. Moreover, different staging methods with great variations lead to easy misunderstandings in many studies and clinical applications. Well-designed studies in the future may help resolve the current disputes and establish universally accepted staging methods.

## 6 Conclusions

Several classification methods with great differences in the SCLNs are used in clinical practice. Anatomically, the deep cervical lymph nodes are the upmost stations, and they can drain lymphatics at various levels of the esophagus, even from the intramural esophagus directly. Cervical metastasis of EC affects mainly the cervical tracheoesophageal nodes and medial SCLNs located on the surface of the scalenus anterior muscle and near the venous angle. These nodes can be commonly found in superficial EC and even become the sentinel nodes. Most studies have suggested that the SCLN metastasis in EC did not have a significant impact on survival, and the survival of patients with SCLN metastasis is similar to those with regional lymph node metastasis and better than those with distant organ metastasis. These clinical results bring challenges to the widely used AJCC and JES staging systems for EC. SCLN metastasis may not be a contraindication of curative surgery, and neoadjuvant chemoradiotherapy followed by surgery still showed the optimal treatment modality for resectable patients (50, 54). Therefore, more well-designed studies are needed in the future to have uniform definition, staging, and treatment of SCLN metastasis in EC.
