# Supplementary material for: Supraclavicular lymph node metastasis in esophageal carcinoma: a topic of ongoing controversy
Source: Front Oncol. 2025 Jan 27;15:1527625. doi: 10.3389/fonc.2025.1527625 (PMC11807800; doi:10.3389/fonc.2025.1527625)
Supplement: Supplementary file 1 [file Table1.docx]

***Supplementary Material***

**Supraclavicular Lymph Node Metastasis in Esophageal Carcinoma: A Topic of Ongoing Controversy**

Bowen Zhang^1 †^, Huan Zhang^1†^, Yu Chen^2^, Wanli Xia^2^, Yichun Wang^1^*

Correspondence: Yichun Wang**,** [wangechun321@sina.com](mailto:wangechun321@sina.com).

**1 Literature search**

**1.1 Search Data: July 21, 2024**

- 1. **Search strategy**

**1.2.1 PubMed**

#1 (((esophageal [Title/Abstract]) OR (oesophageal [Title/Abstract])) OR (esophagus [Title/Abstract])) OR (oesophagus [Title/Abstract])

#2 (((cancer [Title/Abstract]) OR (carcinoma [Title/Abstract])) OR (tumor [Title/Abstract])) OR (neoplasm [Title/Abstract])

#3 1 AND 2

#4 Esophageal cancer [MeSH Terms]

**#5 3 OR 4**

#6 ((supraclavicular [Title/Abstract]) OR (cervical [Title/Abstract])) OR (neck [Title/Abstract])

#7 lymph node [Title/Abstract]

**#8 6 AND 7**

#9 (((three-field [Title/Abstract]) OR (3-field [Title/Abstract])) OR (extensive [Title/Abstract])) OR (extended [Title/Abstract])

#10 (dissection [Title/Abstract]) OR (lymphadenectomy [Title/Abstract])

#11 Lymph Node Excision [MeSH Terms]

#12 10 OR 11

**#13 9 AND 12**

#14 8 OR 13

#15 5 AND 14

**1.2.2 Scopus**

#1 (TITLE-ABS-KEY (“oesophageal”) OR TITLE-ABS-KEY (“esophageal”) OR TITLE-ABS-KEY (“esophagus”) OR TITLE-ABS-KEY (“oesophagus”)) AND (TITLE-ABS-KEY (“cancer”) OR TITLE-ABS-KEY (“carcinoma”) OR TITLE-ABS-KEY (“tumor”) OR TITLE-ABS-KEY (“neoplasm”))

#2 TITLE-ABS-KEY (“supraclavicular”) OR TITLE-ABS-KEY (“cervical”) OR TITLE-ABS-KEY (“neck”) (TITLE-ABS-KEY (“supraclavicular”) OR TITLE-ABS-KEY (“cervical”) OR TITLE-ABS-KEY (“neck”)) AND (TITLE-ABS-KEY (“lymph node”))

#3 (TITLE-ABS-KEY (“three-field “) OR TITLE-ABS-KEY (“3-field”) OR TITLE-ABS-KEY (“extensive”) OR TITLE-ABS-KEY (“extended “)) AND (TITLE-ABS-KEY (“dissection”) OR TITLE-ABS-KEY (“lymphadenectomy “))

#4 #2 OR #3

#5 #1 AND #4

**1.2.3 Web of Science**

**#1 esophageal** (Abstract) or **oesophageal** (Abstract) or **esophagus** (Abstract) or **oesophagus** (Abstract)

**#2**

**cancer** (Abstract) or **carcinoma** (Abstract) or **tumor** (Abstract) or **neoplasm** (Abstract)

**#3 #1 and #2**

#4 **supraclavicular** (Abstract) or **cervical** (Abstract) or **neck** (Abstract)

#5 **lymph node** (Abstract)

**#6 #4 and #5**

**#7**

**three-field** (Abstract) or **3-field** (Abstract) or **extensive** (Abstract) or **extended** (Abstract)

**#8**

**dissection** (Abstract) or **lymphadenectomy** (Abstract)

**#9 #7 and #8**

**#10 #6 or #9**

**#11 #3 and #10**

**2 Relevant studies after systematic search**

**2.1 SCLNs for patients received definitive chemoradiotherapy**

**Table 1.** Prognosis of patients with supraclavicular lymph node metastasis received definitive chemoradiotherapy

| First Author (year) | Staging System | Treatment | Group | Number | SCC/Others | U/M/L | OS | P value |
| --- | --- | --- | --- | --- | --- | --- | --- | --- |
| HY Xu  2018 [1] | 8th AJCC/UICC | dCRT | SCLN (+) | 155 | 143/12 | 45/88/22 | 5-year: 18.5% | <0.001 |
|  |  |  | SCLN (-) | 596 | 567/29 | 158/332/106 | 5-year: 25.1% |  |
| JH Yen  2020 [2] | 8th AJCC/UICC | dCRT | SCLN (+) | 71 | 71/0 | 24/33/14 | 5-year: 11.3% | 0.88 |
|  |  |  | SCLN (-) | 72 | 72/0 | 23/22/27 | 5-year: 15.2% |  |
| H Liu  2011 [3] | 7th AJCC/UICC | dCRT | CLN (-) | 100 | 100/0 | - | mOS: 21m | 0.001 |
|  |  |  | CPLN (+) | 30 | 30/0 | - | mOS: 15m |  |
|  |  |  | OCLN (+) | 78 | 78/0 | - | mOS: 13m |  |
| DK Tong  2008 [4] | 6th AJCC/UICC | CRT+S | SCLN (+) | 22 | 22/0 | - | mOS: 34.8m | <0.001 |
|  |  | dCRT | SCLN (+) | 46 | 46/0 | - | mOS: 9.9m |  |
| WB Shen  2011 [5] | 7th AJCC/UICC | dRT/dCRT | SCLN (+) | 68 | 68/0 | 21/38/9 | mOS: 15.0m | - |
| Y Honma  2017 [6] | 7th AJCC/UICC | S+PT | SCLN (+) | 45 | - | 10/27/8 | 5-year: 43% | 0.138 |
|  |  | S | SCLN (+) | 19 | - | 3/12/4 | 5-year: 46.8% |  |
|  |  | dCRT | SCLN (+) | 38 | - | 7/24/7 | 5-year: 20.5% |  |
| P Zhang  2014 [7] | 6th AJCC/UICC | dCRT | SCLN (+) | 139 | 139/0 | 54/75/10 | 3-year: 27.9% | - |
| YH Chen  2018 [8] | 7th AJCC/UICC | dCRT | Neck LN (+) | 35 | 35/0 | 21/11/3 | mOS: 9.7m | <0.001 |
|  |  |  | Neck LN (-) | 369 | 369/0 | 122/152/95 | mOS: 18.2m |  |
|  |  |  | SCLN (+) | 70 | 70/0 | - | mOS: 17.2m | 0.047 |
|  |  |  | Neck LN (+) | 35 | 35/0 | 21/11/3 | mOS: 9.7m |  |
| X Li  2017 [9] | 7th AJCC/UICC | dCRT/dRT | SCLN (+) | 174 | 174/0 | 70/57/47 | mOS: 19m | 0.785 |
|  |  |  | SCLN (-) | 119 | 119/0 | 30/51/38 | mOS: 17m |  |
| JM Wen  2019 [10] | 6th AJCC/UICC | - | RLN (+) | 323 | - | 323/0/0 | 5-year: 4% | 0.56 |
|  |  |  | SCLN (+) | 27 | - | 27/0/0 | 5-year: 7.4% |  |
|  |  |  | CeLN (+) | 25 | - | 25/0/0 | 5-year: 8% |  |
|  |  |  | RLN (+) | 4723 | - | 0/0/4723 | 5-year: 6.6% | <0.001 |
|  |  |  | SCLN (+) | 588 | - | 0/0/588 | 5-year: 5.8% |  |
|  |  |  | CeLN (+) | 492 | - | 0/0/492 | 5-year: 2.6% |  |
| PM Jeene  2016 [11] | 7th AJCC/UICC | dCRT | SCLN (+) | 37 | - | - | mOS: 23.6 m | 0.51 |
|  |  |  | SCLN (-) | 160 | - | - | mOS: 17.1 m |  |
| YH Chen  2018 [12] | 7th AJCC/UICC | dCRT | SCLN (+) | 70 | 70/0 | - | - | 0.28 |
|  |  |  | SCLN (-) | 299 | 299/0 | - | - |  |
| P Zhang  2014 [13] | 6th AJCC/UICC | dCRT | CLN (-) | 204 | 204/0 |  | mOS: 34 m | <0.001 |
|  |  |  | uCLN (+) | 106 | 106/0 |  | mOS: 27 m |  |
|  |  |  | Other DM | 85 | 85/0 |  | mOS: 23 m |  |

**Abbreviations**: **dCRT:** definitive chemoradiotherapy; **SCC**: squamous cell carcinoma; **U**: upper; **M**: middle; **L**: lower; **SCLN:** supraclavicular lymph node; **uSCLN**: unilateral supraclavicular lymph node; **OS**: overall survival; **mOS**: median overall survival; **LN**: lymph node; **CLN**: cervical lymph node; **CPLN:** cervical paraesophageal lymph node; **OCLN:** other cervical lymph node except cervical paraesophageal lymph node; **CRT+S:** upfront chemoradiotherapy and surgery; **dRT:** definitive radiotherapy; **S+PT**: Surgery plus perioperative therapy; **S**: surgery; **RLN:** regional lymph node; **CeLN:** celiac lymph node; **DM**: distant metastases; m: moths.

**2.2 SCLNs for patients received surgery**

**Table 2.** Prognosis of patients with supraclavicular lymph node metastasis received esophagectomy and lymph node dissection

| First Author (year) | Staging System | Treatment | Group | Number | SCC/  Others | U/M/L | OS | P value |
| --- | --- | --- | --- | --- | --- | --- | --- | --- |
| H Kato  2000 [14] | 4th AJCC/UICC | S with 3FD | CLN (+) | 46 | - | 8/31/7 | 5-year:15.7% | 0.127 |
|  |  |  | MLN (+) | 107 | - | - | - |  |
|  |  |  | CLN (+) | 46 | - | 8/31/7 | 5-year:15.7% | 0.155 |
|  |  |  | ALN (+) | 85 | - | - | - |  |
| S Kosugi  2013 [15] | 7th AJCC/UICC | S with 3FD | SCLN (+) | 6 | - | 2/4/0 | 5-year:46.2% | 0.06 |
|  |  |  | SCLN (-) | 80 | - | 15/55/10 | 5-year:77.8% |  |
| JQ Chen  2014 [16] | 7th AJCC/UICC | S with 3FD | 101 (+) | 219 | 219 | - | 5-year:24.1% | 0.117 |
|  |  |  | 104 (+) | 26 | 26 |  | 5-year:16.2% |  |
|  |  |  | 101+104 (+) | 51 | 51 | - | 5-year:11.7% |  |
| H Igaki  2000 [17] | 5th AJCC/UICC | S with 3FD | CLN (+) | 17 | - | 5/9/3 | 5-year:55% | 0.10 |
|  |  |  | CLN (-) | 84 | - | 10/47/27 | 5-year:71% |  |
| S. Nakagawa  2003 [18] | 5th AJCC/UICC | S with 3FD | CLN (+) | 28 | - | - | 5-year:32.7% | 0.8047 |
|  |  |  | Extra-CLN (+) | 86 | - | - | 5-year:38.7% |  |
| SY Park  2023 [19] | 8th AJCC/UICC | S with 3FD | pN0 | 249 | 249/0 | - | 5-year:64.2% | 0.054 |
|  |  |  | N (+) + SLCN (-) | 287 | 287/0 | - | 5-year:41.5% |  |
|  |  |  | N (+) + SLCN (+) | 75 | 75/0 | - | 5-year:25.6% |  |
| H Miyata  2019 [20] | 7th AJCC/UICC | nCT + S with 2FD or 3FD |  |  | - | - |  |  |
|  |  |  |  |  | - |  |  |  |
| T Nishimaki  1994 [21] | 4th AJCC/UICC |  |  |  |  |  |  |  |
| H Miyata  2015 [22] | 7th AJCC/UICC | nCT + S with 2FD/3FD | N (+) + SLCN (-) | 169 | - | - | 3-year:47.5% | 0.003 |
|  |  |  | N (+) + SLCN (+) | 47 | - | - | 3-year:20.1% |  |
| Y Honma  2017 [6] | 7th AJCC/UICC | DCRT, S, nCT+S |  |  |  |  |  |  |
| FD Wang  2019 [23] | 8th AJCC/UICC | S with 3FD | SCLN (+) | 35 | 35/0 | - | mOS: 21.0 m | <0.001 |
|  |  |  | SCLN (-) | 128 | 128/0 | - | mOS: 39.0m |  |
| KX Li  2024 [24] | 8th AJCC/UICC | S with 3FD | SCLN (+) | 69 | 69/0 | 41/27/1 | 5-year:34.0% | 0.0416 |
|  |  |  | CPLN (+) | 54 | 54/0 | 19/28/6 | 5-year:21.0% |  |
| M Tachibana  2000 [25] | 5th AJCC/UICC | S with 3FD | M1ym | 31 | 31/0 | 4/12/15 | 5-year:12.8% | - |
| Y Sato  2021 [26] | 8th AJCC/UICC | nCRT+S with 3FD | Stage III | 94 | 94/0 | 22/39/33 | 5-year:57.6% | 0.39 |
|  |  |  | IVB with SCLN (+) | 18 | 18/0 | 5/13/0 | 5-year:41.3% |  |
| X Chang  2023 [27] | 7th AJCC/UICC | nCRT+S | SCLN (+) | 23 | - | 0/0/23 | 5-year:22.0% | <0.001 |
|  |  | Upfront s | SCLN (+) | 18 | - | 0/0/18 | 5-year:6.0% |  |
|  |  | dCRT | SCLN (+) | 51 | - | 0/0/51 | 5-year:4.0% |  |
| YK Yu  2022 [28] | 7th AJCC/UICC | nCRT+S | SCLN (+) | 41 | 41/0 | 8/27/6 | 3-year:71.3% | <0.0001 |
|  |  | dCRT | SCLN (+) | 133 | 133/0 | 44/73/16 | 3-year:32.3% |  |
|  |  | RT | SCLN (+) | 23 | 23/0 | 7/14/2 | 3-year:17.4% |  |
| YM Shim  2004 [29] | 6th AJCC/UICC | S with 2/3FLD | M1a | 24 | 24/0 | 9/0/15 | 5-year:23.9% | 0.048 |
|  |  |  | M1b | 29 | 29/0 | 1/23/5 | 5-year:6.1% |  |
| JQ Chen  2014 [30] | 7th AJCC/UICC | S with 3FD | CPLN (+) | 219 | 219/0 | - | 5-year:24.1% | 0.117 |
|  |  |  | SCLN (+) | 26 | 26/0 | - | 5-year:16.2% |  |
|  |  |  | CPLN+SCLN (+) | 51 | 51/0 | - | 5-year:11.7% |  |
| YZ Zheng  2017 [31] | 7th AJCC/UICC | S with 3FD | N1 | 638 | 638/0 | - | 5-year:52.0% | <0.001 |
|  |  |  | N2 | 304 | 304/0 | - | 5-year:35.8% |  |
|  |  |  | N3 | 31 | 31/0 | - | 5-year:19.2% |  |
|  |  |  | SCLN (+) | 183 | 183/0 | - | 5-year:34.6% |  |
| O Shiraishi  2024 [32] | 7th AJCC/UICC | S with 3FD/2FD | 104 (+) | 51 | - | - | 5-year:45.8% | - |
|  |  |  | 101 (+) | 64 | - | - | 5-year:41.6% |  |
|  |  |  | 106Rec (+) | 144 |  |  | 5-year:41.4% |  |
|  |  |  | 107/109 (+) | 86 |  |  | 5-year:34.8% |  |
|  |  |  | 1/2 (+) | 148 |  |  | 5-year:39.1% |  |
|  |  |  | 3/7 (+) | 112 |  |  | 5-year:35.7% |  |
|  |  |  | 8/9/11 (+) | 32 |  |  | 5-year:35.2% |  |
| JM Wen  2019 [10] | 6th AJCC/UICC | - |  |  |  |  |  |  |
|  |  |  |  |  |  |  |  |  |
| Y Numata  2021 [33] | 7th AJCC/UICC | S with 3FD/2FD | N0 | 23 | 20/3 | 23/0/0 | 5-year:58.4% | 0.433 |
|  |  |  | N+ with SLCN (-) | 27 | 25/2 | 27/0/0 | 5-year:46.2% |  |
|  |  |  | N+ with SLCN (+) | 17 | 16/1 | 17/0/0 | 5-year:7.2% | <0.001 |
|  |  |  | N+ with SLCN (-) | 27 | 25/2 | 27/0/0 | 5-year:46.2% |  |
| S Igaue  2024 [34] | 8th AJCC/UICC | nCT+S with 3FD | M0 | 79 | 69/10 | 18/31/30 | - | 0.721 |
|  |  |  | rM1LN | 80 | 72/8 | 26/28/26 | 3-year:76.7% |  |
| A Okamura  2018 [35] | 8th AJCC/UICC | S with 3FD | N0 | 94 | 94/0 | 0/94/0 | 5-year:50.0% | 0.002 |
|  |  |  | N+ | 78 | 78/0 | 0/78/0 | 5-year:47.4% |  |
|  |  |  | SCLN/CELN (+) | 38 | 38/0 | 0/38/0 | 5-year:21.1% | 0.001 |
|  |  |  | N+ | 78 | 78/0 | 0/78/0 | 5-year:47.4% |  |
| WK Cho  2016 [36] | 6th AJCC/UICC | nCRT+S with 3FD | M0 | 104 | 104/0 | - | 2-year:67.4% | 0.515 |
|  |  |  | SCLN/CELN (+) | 64 | 64/0 | - | 2-year:69.3% |  |
| Y Tachimori  2014 [37] | 7th AJCC/UICC | S with 3FD | N0 | 559 | - | - | 5-year:73,7% | <0.001 |
|  |  |  | N (+) + SLCN (-) | 560 | - | - | 5-year:40.4% |  |
|  |  |  | N (+) + SLCN (-) | 560 | - | - | 5-year:40.4% | <0.001 |
|  |  |  | N (+) + SLCN (+) | 190 | - | - | 5-year:24.1% |  |
| W. Hu [38] | 7th AJCC/UICC | S with 3FD | SCLN (+) | 72 | 72/0 | 53/16/3 | 5-year:24.0% | 0.000 |
|  |  |  | SCLN (-) | 204 | 204/0 | 115/60/29 | 5-year:59.2% |  |

**Abbreviations**: **SCLN**, supraclavicular lymph node; **CLN**, cervical lymph node; **MLN**, mediastinal lymph node; **ALN,** abdominal lymph node; **M1ym**, the cervical and celiac lymph node metastases; **U,** upper EC; **M,** Middle EC; **L,** lower EC; **LN,** lymph node; **LLNM,** Local lymph node metastases; **SC,** supraclavicular; **CE**, celiac; **dCRT**, definitive chemoradiotherapy; **nCRT,** neoadjuvant chemoradiotherapy; **2FD,** two-field dissection; **3FD,** three-field dissection; **S,** Surgery; **RT,** radiotherapy; **m**, moths; **mOS**, median overall survival.

**3 References**

1. Xu HY, Wu SX, Luo HS, Chen CY, Lin LX, Huang HC: **Analysis of definitive chemo-radiotherapy for esophageal cancer with supra-clavicular node metastasis based on CT in a single institutional retrospective study: A propensity score matching analysis**. *BMC Emergency Medicine* 2018, **18**(1).

2. Yen JH, Jen CW, Huang TT, Tsai YC, Cheng SHC: **Association of supraclavicular node metastasis with survival in node positive esophageal squamous cell carcinoma patients treated using definitive chemoradiation**. *Therapeutic Radiology and Oncology* 2020, **4**.

3. Liu H, Lu L, Zhu Q, Hao Y, Mo Y, Liu M, Hu Y, Cui N, Rong T: **Cervical nodal metastases of unresectable thoracic esophageal squamous cell carcinoma: Characteristics of long-term survivors after concurrent chemoradiotherapy**. *Radiotherapy and Oncology* 2011, **99**(2):181-186.

4. Tong DKH, Kwong DLW, Law S, Wong KH, Wong J: **Cervical nodal metastasis from intrathoracic esophageal squamous cell carcinoma is not necessarily an incurable disease**. In: *Journal of Gastrointestinal Surgery: 2008*; 2008: 1638-1645.

5. Shen W, Zhu S, Wan J, Li S, Zhang C, Su J, Li J, Liu Z, Li Y: **Clinical analysis of 3-dimensional conformal radiotherapy for esophageal carcinoma with supraclavicular lymph node metastasis**. *Chinese Journal of Clinical Oncology* 2011, **38**(4):218-221+224.

6. Honma Y, Hokamura N, Nagashima K, Sudo K, Shoji H, Iwasa S, Takashima A, Kato K, Hamaguchi T, Boku N *et al*: **Clinical outcomes of resectable esophageal cancer with supraclavicular lymph node metastases treated with curative intent**. *Anticancer Research* 2017, **37**(7):3741-3749.

7. Zhang P, Xi M, Zhao L, Li QQ, He LR, Liu SL, Shen JX, Liu MZ: **Efficacy and prognostic analysis of chemoradiotherapy in patients with thoracic esophageal squamous carcinoma with cervical lymph nodal metastasis alone**. *Radiation oncology (London, England)* 2014, **9**:256.

8. Chen YH, Lu HI, Lo CM, Wang YM, Chou SY, Hsiao CC, Shih LH, Chen SW, Li SH: **Neck Lymph Node Metastasis as A Poor Prognostic Factor in Thoracic Esophageal Squamous Cell Carcinoma Patients Receiving Concurrent Chemoradiotherapy: A Propensity Score-Matched Analysis**. *Scientific Reports* 2018, **8**(1).

9. Li X, Zhao L, Zhang W, Yang C, Lian Z, Wang S, Liu N, Pang Q, Wang P, Yu J: **Prognostic value of supraclavicular nodes and upper abdominal nodes metastasis after definitive chemoradiotherapy for patients with thoracic esophageal squamous cell carcinoma**. *Oncotarget* 2017, **8**(39):65171-65185.

10. Wen J, Chen D, Zhao T, Chen J, Zhao Y, Liu D, Wang W, Xu X, Fan M, Chen C, Chen Y: **Should the clinical significance of supraclavicular and celiac lymph node metastasis in thoracic esophageal cancer be reevaluated?** *Thoracic Cancer* 2019, **10**(8):1725-1735.

11. Jeene PM, Versteijne E, van Berge Henegouwen MI, Bergmann JJGHM, Geijsen ED, van Laarhoven HWM, Hulshof MCCM: **Supraclavicular node disease is not an independent prognostic factor for survival of esophageal cancer patients treated with definitive chemoradiation**. *Acta Oncologica* 2017, **56**(1):33-38.

12. Chen YH, Lu HI, Lo CM, Wang YM, Chou SY, Huang CH, Shih LH, Chen SW, Li SH: **The clinical impact of supraclavicular lymph node metastasis in patients with locally advanced esophageal squamous cell carcinoma receiving curative concurrent chemoradiotherapy**. *PLoS ONE* 2018, **13**(6).

13. Zhang P, Xi M, Zhao L, Li QQ, He L, Liu S, Shen J, Liu MZ: **Unilateral cervical nodal metastasis is an independent prognostic factor for esophageal squamous cell carcinoma patients undergoing chemoradiotherapy: A retrospective study**. *PLoS ONE* 2014, **9**(6).

14. Kato H, Igaki H, Tachimori Y, Watanabe H, Tsubosa Y, Nakanishi Y: **Assessment of cervical lymph node metastasis in the staging of thoracic esophageal carcinoma**. *Journal of Surgical Oncology* 2000, **74**(4):282-285.

15. Kosugi SI, Kawaguchi Y, Kanda T, Ishikawa T, Sakamoto K, Akaike H, Fujii H, Wakai T: **Cervical lymph node dissection for clinically submucosal carcinoma of the thoracic esophagus**. *Annals of Surgical Oncology* 2013, **20**(12):4016-4021.

16. Chen J, Wu S, Zheng X, Pan J, Zhu K, Chen Y, Li J, Liao L, Lin Y, Liao Z: **Cervical lymph node metastasis classified as regional nodal staging in thoracic esophageal squamous cell carcinoma after radical esophagectomy and three-field lymph node dissection**. *BMC Surgery* 2014, **14**(1).

17. Igaki H, Kato H, Tachimori Y, Nakanishi Y: **Cervical lymph node metastasis in patients with submucosal carcinoma of the thoracic esophagus**. *Journal of Surgical Oncology* 2000, **75**(1):37-41.

18. Nakagawa S, Nishimaki T, Kosugi S, Ohashi M, Kanada T, Hatakeyama K: **Cervical lymphadenectomy is beneficial for patients with carcinoma of the upper and mid-thoracic esophagus**. *Diseases of the Esophagus* 2003, **16**(1):4-8.

19. Park SY, Lee J, Jeon YJ, Cho JH, Kim HK, Choi YS, Zo JI, Shim YM: **Clinical and Pathologic Supraclavicular Lymph Node Metastases in Esophageal Squamous Cell Carcinoma Treated by Esophagectomy with Three-Field Lymph Node Dissection**. *Annals of Surgical Oncology* 2024, **31**(5):3399-3408.

20. Miyata H, Sugimura K, Yamasaki M, Makino T, Tanaka K, Morii E, Omori T, Yamamoto K, Yanagimoto Y, Yano M *et al*: **Clinical Impact of the Location of Lymph Node Metastases After Neoadjuvant Chemotherapy for Middle and Lower Thoracic Esophageal Cancer**. *Annals of Surgical Oncology* 2019, **26**(1):200-208.

21. Nishimaki T, Tanaka O, Suzuki T, Aizawa K, Hatakeyama K, Muto T: **Clinical implications of cervical lymph node metastasis patterns in thoracic esophageal cancer**. *Annals of Surgery* 1994, **220**(6):775-781.

22. Miyata H, Yamasaki M, Miyazaki Y, Takahashi T, Kurokawa Y, Nakajima K, Takiguchi S, Mori M, Doki Y: **Clinical importance of supraclavicular lymph node metastasis after neoadjuvant chemotherapy for esophageal squamous cell carcinoma**. *Annals of Surgery* 2015, **262**(2):280-285.

23. Wang F, Ge X, Wang Z, Weng Y, Yin R, You Q: **Clinical significance and prognosis of supraclavicular lymph node metastasis in patients with thoracic esophageal cancer**. *Ann Transl Med* 2020, **8**(4):90.

24. Li K, Li C, Lu S, He W, Wang C, Han Y, Leng X, Peng L: **Effect of cervical paraesophageal lymph node metastasis versus supraclavicular lymph node metastasis on the overall survival of patients with thoracic esophageal squamous cell carcinoma: an observational study**. *Ann Med Surg (Lond)* 2024, **86**(5):2518-2523.

25. Tachibana M, Dhar DK, Kinugasa S, Kotoh T, Shibakita M, Ohno S, Masunaga R, Kubota H, Nagasue N: **Esophageal cancer with distant lymph node metastasis: Prognostic significance of metastatic lymph node ratio**. *Journal of Clinical Gastroenterology* 2000, **31**(4):318-322.

26. Sato Y, Motoyama S, Wada Y, Wakita A, Kawakita Y, Nagaki Y, Terata K, Imai K, Anbai A, Hashimoto M, Minamiya Y: **Neoadjuvant chemoradiotherapy followed by esophagectomy with three-field lymph node dissection for thoracic esophageal squamous cell carcinoma patients with clinical stage iii and with supraclavicular lymph node metastasis**. *Cancers* 2021, **13**(5):1-14.

27. Chang X, Liu J, Zhao Y, Shi A, Yu H, Yu R, Wang W: **Neoadjuvant chemoradiotherapy followed by oesophagectomy may be the optimal treatment option for lower thoracic oesophageal cancer with supraclavicular lymph node metastasis: An inverse probability of treatment-weighted analysis of SEER database**. *Journal of Medical Imaging and Radiation Oncology* 2023, **67**(6):676-683.

28. Yu Y, Xu L, Chen X, Li H, Liu Q, Zhang R, Xie H, Chen Y, Yuan L, Tan B *et al*: **Neoadjuvant therapy combined with surgery is superior to chemoradiotherapy in esophageal squamous cell cancer patients with resectable supraclavicular lymph node metastasis: a propensity score-matched analysis**. *Ann Transl Med* 2022, **10**(6):349.

29. Young MS, Yong SC, Kim K: **Prognosis after surgical resection of M1a/M1b esophageal squamous cell carcinoma**. *Journal of Korean Medical Science* 2005, **20**(2):229-231.

30. Chen J, Zhu K, Zheng X, Chen M, Lin Y, Pan C, Pan J: **Prognostic analysis of cervical lymph node metastasis in patients with thoracic esophageal squamous cell carcinoma**. *Chinese Journal of Oncology* 2014, **36**(8):612-616.

31. Zheng Y, Wang Z, Wang F, Huang Q, Liu S: **Proposed modifications of supraclavicular lymph node metastasis in the esophageal squamous cell carcinoma staging system for improved survival stratification**. *Oncotarget* 2017, **8**(25):41563-41571.

32. Shiraishi O, Hagi T, Hiraki Y, Kato H, Koda M, Nakanishi T, Yasuda A, Shinkai M, Imano M, Yasuda T: **Risk factors and prognosis for supraclavicular lymph node metastasis in patients with thoracic esophageal cancer. Distant or regional metastasis?** *Dis Esophagus* 2024.

33. Numata Y, Abe T, Higaki E, Hosoi T, Fujieda H, Nagao T, Hanai N, Suzuki H, Nishikawa D, Matsuo K *et al*: **Should the Supraclavicular Lymph Nodes be Considered Regional Lymph Nodes in Cervical Esophageal Cancer?** *Annals of Surgical Oncology* 2022, **29**(1):616-626.

34. Igaue S, Nozaki R, Utsunomiya D, Kubo Y, Kubo K, Kurita D, Yamamoto S, Ishiyama K, Oguma J, Kato K, Daiko H: **Significance of Surgery for Resectable M1 Lymph Node Metastases Without Organ Metastasis in Esophageal Carcinoma in the Era of Neoadjuvant Treatment**. *Annals of Surgical Oncology* 2024, **31**(3):1525-1535.

35. Okamura A, Watanabe M, Kozuki R, Toihata T, Yuda M, Imamura Y, Mine S: **Supraclavicular and celiac metastases in squamous cell carcinoma of the middle thoracic esophagus**. *Langenbeck's Archives of Surgery* 2018, **403**(8):977-984.

36. Cho WK, Oh D, Ahn YC, Shim YM, Zo JI, Sun JM, Ahn MJ, Park K: **Supraclavicular and/or celiac lymph node metastases from thoracic esophageal squamous cell carcinoma did not compromise survival following neoadjuvant chemoradiotherapy and surgery**. *Oncotarget* 2017, **8**(2):3542-3552.

37. Tachimori Y, Ozawa S, Numasaki H, Matsubara H, Shinoda M, Toh Y, Udagawa H: **Supraclavicular node metastasis from thoracic esophageal carcinoma: A surgical series from a Japanese multi-institutional nationwide registry of esophageal cancer**. *Journal of Thoracic and Cardiovascular Surgery* 2014, **148**(4):1224-1229.

38. Hu W, Liu J, He F, Wang C, Ma Z: **Supraclavicular node metastasis in thoracic esophageal squamous cell carcinoma**. *European Surgery - Acta Chirurgica Austriaca* 2014, **46**(4):139-143.
